# Overexpression of the Transcription Factors GmSHN1 and GmSHN9 Differentially Regulates Wax and Cutin Biosynthesis, Alters Cuticle Properties, and Changes Leaf Phenotypes in Arabidopsis

**DOI:** 10.3390/ijms17040587

**Published:** 2016-04-21

**Authors:** Yangyang Xu, Hanying Wu, Mingming Zhao, Wang Wu, Yinong Xu, Dan Gu

**Affiliations:** 1 Key Laboratory of Photobiology, Institute of Botany, Chinese Academy of Sciences, Beijing 100093, China; yyxu@ibcas.ac.cn (Ya.X.); whywu@ibcas.ac.cn (H.W.); zhaomm@bioyong.com (M.Z.); wuwang19@163.com (W.W.); yinongxu@ibcas.ac.cn (Yi.X.); 2 College of Life Sciences, University of Chinese Academy of Sciences, Beijing 100049, China

**Keywords:** wax, cutin, SHN/WIN, AP2/ERF, soybean, overexpression, transcription factor, leaf development

## Abstract

SHINE (SHN/WIN) clade proteins, transcription factors of the plant-specific APETALA 2/ethylene-responsive element binding factor (AP2/ERF) family, have been proven to be involved in wax and cutin biosynthesis. *Glycine max* is an important economic crop, but its molecular mechanism of wax biosynthesis is rarely characterized. In this study, 10 homologs of Arabidopsis *SHN* genes were identified from soybean. These homologs were different in gene structures and organ expression patterns. Constitutive expression of each of the soybean *SHN* genes in Arabidopsis led to different leaf phenotypes, as well as different levels of glossiness on leaf surfaces. Overexpression of *GmSHN1* and *GmSHN9* in Arabidopsis exhibited 7.8-fold and 9.9-fold up-regulation of leaf cuticle wax productions, respectively. C31 and C29 alkanes contributed most to the increased wax contents. Total cutin contents of leaves were increased 11.4-fold in *GmSHN1* overexpressors and 5.7-fold in *GmSHN9* overexpressors, mainly through increasing C16:0 di-OH and dioic acids. *GmSHN1* and *GmSHN9* also altered leaf cuticle membrane ultrastructure and increased water loss rate in transgenic Arabidopsis plants. Transcript levels of many wax and cutin biosynthesis and leaf development related genes were altered in *GmSHN1* and *GmSHN9* overexpressors. Overall, these results suggest that *GmSHN1* and *GmSHN9* may differentially regulate the leaf development process as well as wax and cutin biosynthesis.

## 1. Introduction

Higher plants have developed an extracellular hydrophobic cuticle layer that covers their aerial epidermis, providing protection against nonstomatal water loss and various forms of biotic and abiotic stresses [1,2,3,4]. Cuticle is a heterogeneous layer consists of cutin polyester matrix covered with epicuticular waxes and filled with intracuticular waxes [5,6]. Cuticular waxes, mainly composed of very-long-chain alkanes, fatty acids, primary and secondary alcohols, esters, aldehydes, and ketones, are responsible for the glossy appearances in leaves and fruits [7]. Wax biosynthesis begins at the endoplasmic reticulum (ER) with C16 and C18 fatty acids as precursors, followed by the fatty acid elongation process through which fatty acids are converted into very-long-chain fatty acids (VLCFAs). VLCFAs are converted into other wax components through two pathways. One is the alkane forming pathway that yields aldehydes, alkanes, secondary alcohols, and ketones; the other one is the alcohol forming pathway that produces primary alcohols and esters [8].

The coordinated expressions of multiple related genes are involved in the biosynthesis of cuticular waxes [7,9], and these genes are transcriptionally mediated by transcription factors through specific recognition of trans-acting factors and *cis*-acting elements [10,11]. The AP2/ERF family, a plant-specific transcription factor superfamily that is involved in various events during plant growth and development, has been reported to act as regulators in wax biosynthesis [12,13,14].

SHINE (SHN/WIN) clade proteins belong to the APETALA 2/ethylene-responsive element binding factor (AP2/ERF) superfamily and have been widely reported to meditate wax biosynthesis in Arabidopsis [15,16], rice [17,18], wheat [19], tomato [20], and apple [21]. In Arabidopsis, *SHN* genes encode a small distinct clade of three proteins which share a highly conserved AP2 domain and two other conserved motifs [15]. Overexpression of either one of Arabidopsis *SHN* genes induces wax accumulation and leads to glossy appearances on the plant surface [16,22]. Silencing of *SHN* clade genes in Arabidopsis was shown to reduce flower cutin load, modify petal cell wall structure, and result in the alternation of floral organ morphology and surface formation [23]. Furthermore, *AtSHN1* was also reported to confer drought tolerance [15] and improve defense resistance when overexpressed in plants [24]. Recently, Al-Abdallat proposed that overexpression of the *SlSHN1* gene improves drought tolerance by increasing cuticular wax accumulation in tomato [10]. These results indicate that *SHN* genes play important roles in plants.

Soybean (*Glycine max* L.) is an important leguminous crop that provides abundant protein and oil for food production and animal forage. Genome sequencing of soybeans has provided great convenience for addressing functions of important genes. Although a variety of studies on *SHN* genes have been made in plants, the characterization of soybean *SHN* genes is rarely investigated. Previous studies showed that soybeans have undergone two separate polyploidy events [25], and postulated that phenotypic variation may be derived from differential expression of duplicated genes, especially in polyploids [26]. The identification and functional characterization of soybean *SHN* genes may provide more information on the SHN transcription factors. Also, the fundamental researches might be useful for further improvement of soybean crop production.

In this study, we identified 10 homologs of Arabidopsis *SHN* genes in soybeans and analyzed their expression patterns, as well as their functions through heterologous overexpression. These homologs were differentially expressed in various soybean organs, and overexpression of each soybean *SHN* homologs in Arabidopsis led to different levels of glossiness on leaf surfaces, as well as different leaf shapes and colors. Overexpression of *GmSHN1* and *GmSHN9* in Arabidopsis showed increased leaf wax and cutin accumulation, altered expression of wax and cutin biosynthesis and leaf development related genes, and altered leaf cuticle ultrastructure and permeability. Our results are the first, to our knowledge, to focus on the differential expression of SHN transcription factors in the wax and cutin biosynthesis pathway as well as leaf development process. This finding provides new insight into SHN-mediated plant development, and enriches information about the SHN transcription factor family in various species.

## 2. Results

### 2.1. Isolation and Identification of Soybean SHINE Homologs

**Figure 1:**
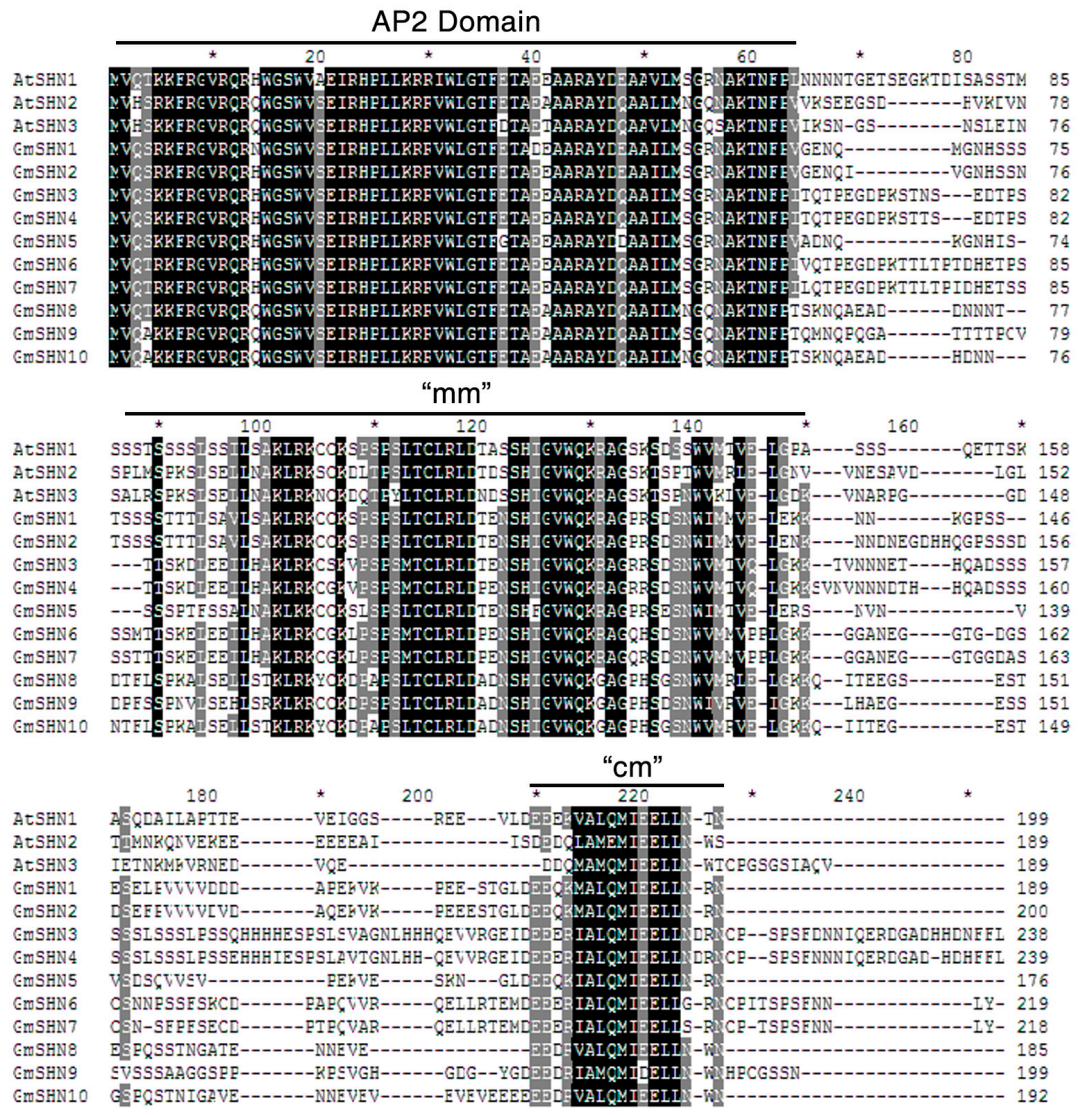
Multiple sequence alignment of the three Arabidopsis SHN proteins and ten putative homologs from soybean. Residues are highlighted in black for 100% identical, gray for at least 80% identical, respectively. mm, middle motif; cm, C-terminal motif; *, abbreviation of sequence numbers.

Database-searching that use Arabidopsis SHN1/SHN2/SHN3 amino acid sequences as probes led to the identification of 10 putative soybean SHN homologs. The sequence lengths of soybean SHN proteins range from 176 to 239 amino acids, and all the proteins contain the conserved AP2 domain (Figure 1). In addition, soybean SHN proteins share two other conserved motifs, middle motif (mm) and C-terminal motif (cm), with Arabidopsis SHN clade members. Genes encoding these homologs in soybean were designated as *GmSHN1* to *GmSHN10* based on amino acid sequence similarity score to AtSHN1: *GmSHN1* (Glyma07g03500), *GmSHN2* (Glyma08g22590), *GmSHN3* (Glyma13g23570), *GmSHN4* (Glyma17g12330), *GmSHN5* (Glyma15g01140), *GmSHN6* (Glyma06g29110), *GmSHN7* (Glyma04g19650), *GmSHN8* (Glyma06g07240), *GmSHN9* (Glyma17g31900) and *GmSHN10* (Glyma04g07140). The alignment of amino acid sequences suggests that GmSHN1 shows the highest sequence similarity to the AtSHN1 probe (62.4% of identity); GmSHN10 shows the highest sequence similarity to both the AtSHN2 (63.6% of identity) and AtSHN3 probes (62.3% of identity); GmSHN10 shows the lowest sequence similarity to the AtSHN1 probe (57.9% of identity); GmSHN7 shows the lowest sequence similarity to the AtSHN2 probe (51.9% of identity); and GmSHN4 shows the lowest sequence similarity to the AtSHN3 probe (49.5% of identity).

**Figure 2:**
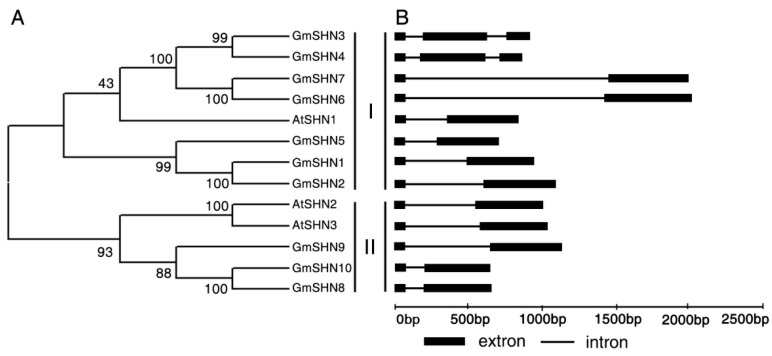
Sequence and gene structure analysis of soybean SHN homologs with Arabidopsis SHN proteins. (**A**) Phylogenetic analysis of 10 soybean SHN proteins and 3 Arabidopsis SHN proteins. The neighbor-joining tree was constructed using the MEGA 6.0 software (Availeble at: http://www.megasoftware.net/). The accession numbers of Arabidopsis are as follows: AtSHN1 (AT1G15360), AtSHN2 (AT5G11190), and AtSHN3 (AT5G25390); (**B**) Gene structures analysis of *SHN* genes. Extrons are shown as black boxes and introns are shown as lines.

The phylogenetic tree showed that 10 GmSHN proteins can be classified into two clusters based on the neighbor-joining method. While cluster I contains GmSHN1 to GmSHN7, cluster II consists of GmSHN8, GmSHN9, and GmSHN10 (Figure 2A). We found some pairwise GmSHN proteins with high sequence similarity, GmSHN1 and GmSHN2 (89.4% identity), GmSHN3 and GmSHN4 (89.4% identity), GmSHN6 and GmSHN7 (89.4% identity), and GmSHN8 and GmSHN10 (93.4% identity), respectively. These pairwise proteins might share a common ancestor and similar biological functions.

Furthermore, analysis of gene structure shows that all *GmSHN* genes have their first intron position at 80 bp from the start codon (Figure 2B). These results are consistent with gene structures of *AtSHN1/SHN2/SHN3*. *GmSHN6* and *GmSHN7* contain the longest introns among the 10 *GmSHN* genes. *GmSHN3* and *GmSHN4* comprise three extrons and harbor two introns in their coding regions, whereas other soybean *SHN* genes contain a single intron. The differences in gene structure might confer functional variations.

### 2.2. GmSHN Genes Are Differentially Expressed in Various Organs of Soybean

**Figure 3:**
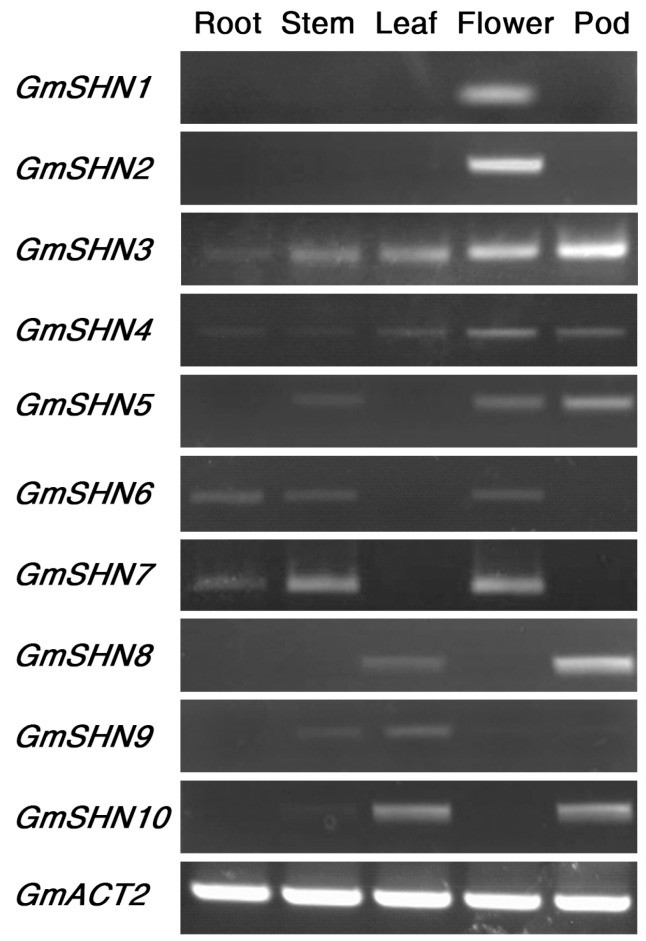
Organ expression patterns of soybean *SHN* genes. All 10 members of soybean *SHN* genes were analyzed by reverse transcription polymerase chain reaction (RT-PCR) in various organs from soybean plants. *GmACT2* was used as control.

To gain insight into the possible functions of soybean SHN proteins, we first investigated the expression patterns of *GmSHN* genes in various organs of adult soybean plants by semi-quantitative reverse transcription polymerase chain reaction (RT-PCR). As shown in Figure 3, the expression of *GmSHN1* and *GmSHN2* could be detected only in floral organs, and the transcript levels of *GmSHN3* and *GmSHN4* were higher in flowers and pods than in other organs. *GmSHN5* had weak expression in stems, flowers, and pods. The mRNA levels of *GmSHN6* and *GmSHN7* were also notably higher in floral organs than that in adult stems and roots. The highest transcript levels of *GmSHN8* and *GmSHN10* were in pods, followed by leaves. *GmSHN9* had weak expression in adult stems and leaves. We performed three independent assays with RNA isolated from different samples of adult soybean plants and received similar results each time. These results suggest that the 10 soybean *SHN* genes were differentially expressed in various soybean organs.

### 2.3. Constitutive Expression of GmSHN Genes in Arabidopsis Results in Different Leaf Phenotypes

**Figure 4:**
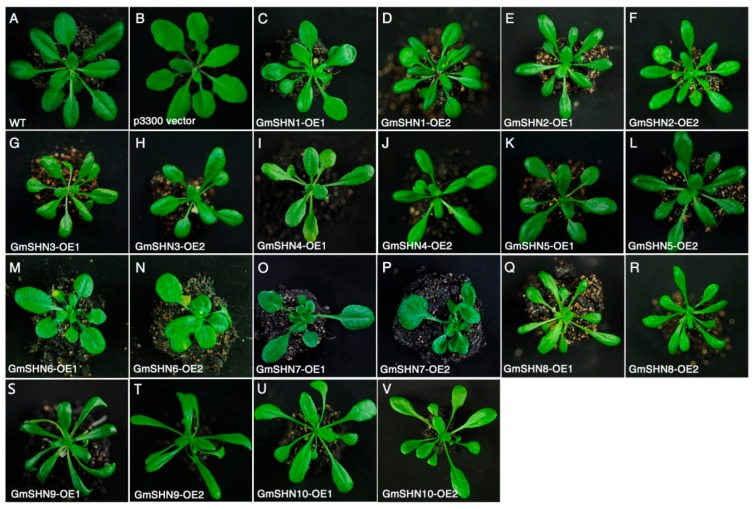
The constitutive expression of *GmSHN* genes in Arabidopsis plants. WT (**A**) and a transgenic line overexpressing the p3300 empty vector (**B**) were used as negative controls. Transgenic lines overexpressing each of the *GmSHN* genes (**C**–**V**) showed differences in leaf phenotypes. Pictures were taken at the same magnification and all the plants were at the period of four weeks old.

We screened the Arabidopsis transgenic lines that individually overexpress each of the 10 *GmSHN* genes and obtained over 20 independent homozygous T3 lines for each *GmSHN* gene. RT-PCR was subsequently performed to confirm the expression of *GmSHN* genes in Arabidopsis. According to the overall morphology, overexpressors of *GmSHN* genes exhibited different levels of glossiness on surfaces of rosette leaves, as well as different leaf shapes and colors (Figure 4). Phenotypes of *GmSHN* overexpressors can be divided into two groups based on leaf appearances and shapes. Transgenic lines of *GmSHN1*, *GmSHN2*, *GmSHN3*, *GmSHN4*, *GmSHN5*, *GmSHN6,* and *GmSHN7* were similar to the phenotypes produced by overexpression of *AtSHN1* in Arabidopsis [15,16] and exhibited smaller and shiny leaves. Leaf indexes (leaf length/leaf width ratio) [27] of these transgenic lines were similar to that of wild type (WT). However, transgenic lines of *GmSHN8*, *GmSHN9,* and *GmSHN10* exhibited yellow-green, curly, and more slender leaves with longer petioles, which have never been reported in previous *SHN* related studies. Leaf indexes of *GmSHN8*, *GmSHN9,* and *GmSHN10* overexpressors are higher than that of WT.

**Figure 5:**
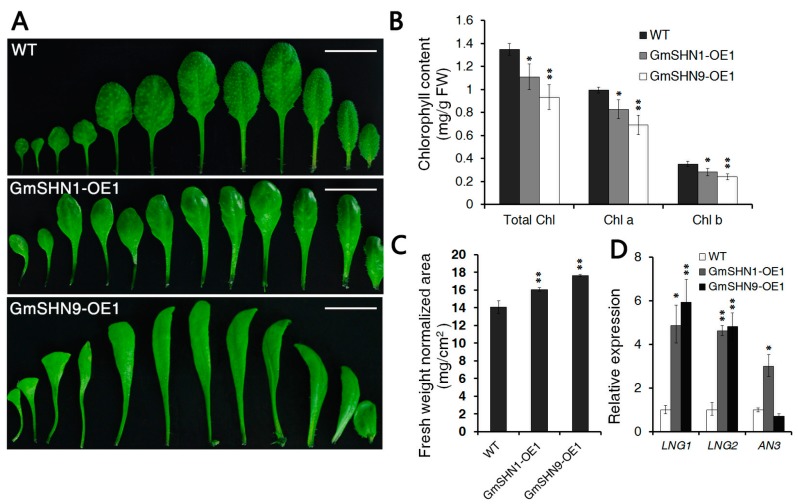
Morphological and biological analysis of transgenic plants. (**A**) Anatomy analysis of 4-week-old Arabidopsis rosette leaves of WT, GmSHN1-OE1 and GmSHN9-OE1. GmSHN1-OE1 had smaller leaves, and GmSHN9-OE1 exhibited yellow-green, longer, and more slender leaves. We performed three independent assays and received similar results each time; (**B**) Analysis of total chlorophyll (total Chl), chlorophyll a (Chl a) and chlorophyll b (Chl b) in GmSHN1-OE1, GmSHN9-OE1, and WT leaves. Presented values are means from three independent replicates (±SD); (**C**) Analysis of leaf fresh weight normalized area of GmSHN1-OE1, GmSHN9-OE1, and WT. Leaf fresh weight normalized area (mg/cm^2^) increased 14% in GmSHN1-OE1 and 25% in GmSHN9-OE1. The data are given as mean values with ±SD (*n* = 3); (**D**) Expression of leaf development related genes. Expression levels of genes in WT were taken as 1. The Arabidopsis *ACT2* gene was used as the internal control for normalization of the template cDNAs. The results are given as mean values of three replicates, and error bars represent SD (*n* = 3). * *p* < 0.05, ** *p* < 0.01 *vs.* WT with two tailed Student’s *t* test.

Based on differences in leaf phenotypes and leaf indexes of *GmSHN* ovexpressors described above, we chose transgenic lines of *GmSHN1* and *GmSHN9* from each group as materials for further study. An anatomical analysis of 4-week-old rosette leaves of GmSHN1-OE1, GmSHN9-OE1, and wild-type showed more detailed differences in leaf phenotypes (Figure 5A). GmSHN1-OE1 and GmSHN9-OE1 both exhibited reduced trichomes and increased glossiness on leaf surfaces. GmSHN9-OE1 exhibited noticeably longer and more slender leaves than WT. The numbers of rosette leaves from GmSHN1-OE1 and GmSHN9-OE1 remained the same with WT plants.

To further explore the changes of leaf color in GmSHN1-OE1 and GmSHN9-OE1, chlorophyll contents were examined and compared with wild-type leaves. The overexpression of *GmSHN1* and *GmSHN9* significantly reduced the total chlorophyll (Chl a and Chl b) pigment contents in the leaves of transgenic plants (Figure 5B). We also examined the leaf fresh weight normalized area of GmSHN1-OE1, GmSHN9-OE1, and WT. Our results showed that leaf fresh weight normalized area increased 14% in GmSHN1-OE1 (16.05 mg/cm^2^, fresh weight) and 25% in GmSHN9-OE1 (17.63 mg/cm^2^, fresh weight) when compared with wild-type (Figure 5C). These results were consistent with the phenotypes of thicker leaves. To further explore the alteration of leaf morphology, we further investigated the expression of nine genes that are involved in leaf development. *AtLNG1* and *AtLNG2* [28] that participate in the elongation of plant leaves were significantly activated in the two transgenic plants (Figure 5B). *AtAN3*, which is reported to control the leaf-width cell proliferation [29], was increased in GmSHN1-OE1 but decreased in GmSHN9-OE9. Transcript levels of other genes did not show significant changes in GmSHN9-OE1 and GmSHN9-OE9 (Figure S1).

### 2.4. GmSHN1 and GmSHN9 Proteins Are Localized Primarily in the Nucleus

**Figure 6:**
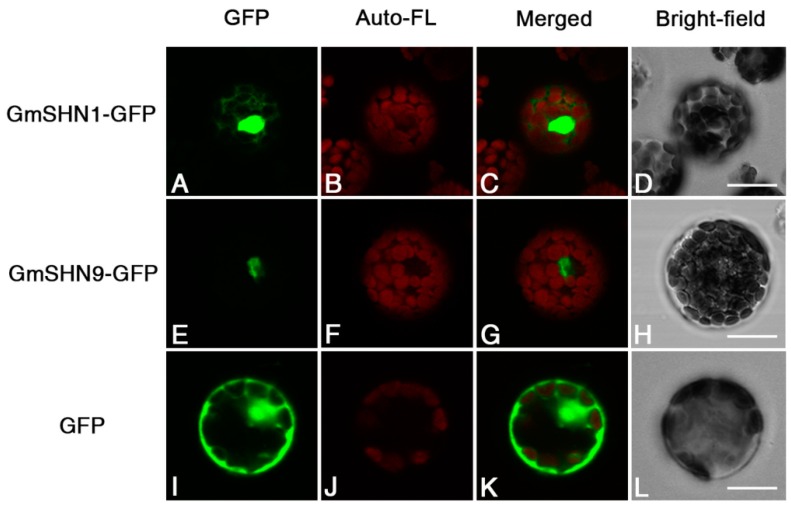
Subcellular localizations of GmSHN1 and GmSHN9. The green florescent protein (GFP) signals obtained by confocal microscopy indicated that fusion proteins GFP-GmSHN1 and GFP-GmSHN9 were localized primarily in the nucleus (**A**,**E**); and GFP protein was observed in both nucleus and cytosol (**I**); The chlorophyll auto fluorescent signals of protoplasts are indicated in images (**B**,**F**,**J**); The overlap of green fluorescent and chlorophyll auto fluorescent signals are indicated in merged images (**C**,**G**,**K**); Bright fields are showed in images (**D**,**H**,**L**). Scale bar = 20 µm.

Nuclear localization is necessary for the function of transcription factor. To test the possibility that GmSHN1 and GmSHN9 act as transcription factors, subcellular localizations of GmSHN1 and GmSHN9 were firstly predicted with Plant-mPLoc software [30], and were further confirmed through Arabidopsis protoplast transient transformation. We constructed fusion proteins of GmSHN1 and GmSHN9 with green florescent protein (GFP). Constructs (GmSHN1-GFP, GmSHN9-GFP, and GFP) were introduced to Arabidopsis protoplast, respectively. As shown in Figure 6, transient expression analysis in protoplast showed that GmSHN1 and GmSHN9 proteins were localized primarily in the nucleus, while GFP was observed in both nucleus and cytosol. We performed three independent assays and received similar results each time. These results further suggest that GmSHN1 and GmSHN9 act as transcription factors.

### 2.5. Overxpression of GmSHN1 and GmSHN9 Induces Epicuticular Wax Accumulation in Transgenic Arabidopsis Leaves

**Figure 7:**
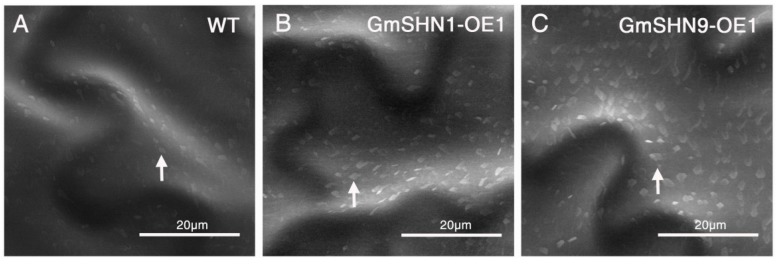
Environmental scanning electron microscopy (ESEM) analysis of leaf cuticle wax depositions. Adaxial side of 4-week-old Arabidopsis rosette leaves of WT, GmSHN1-OE1, and GmSHN9-OE1 were observed under 4000× magnification. (**A**) WT showed only little wax deposition on Leaf surface. The leaf surfaces of transgenic plants GmSHN1-OE1 (**B**); and GmSHN9-OE1 (**C**) are covered with regions of high wax deposition. Wax crystals were shown by arrows.

Previous studies showed that overexpression of *AtSHN* genes alters epicuticular waxes content and component in Arabidopsis. To investigate whether the glossy and shiny leaves of the overexpressors were caused by an alteration in cuticular wax deposition, the adaxial side of 6-week-old rosette leaves of GmSHN1-OE1, GmSHN9-OE1, and WT were observed by environmental scanning electron microscopy (ESEM). As shown in Figure 7, depositions of the epidermal wax crystals were different between the overexpressors and WT. Epidermal surface of WT leaves showed only little wax deposition. The leaf epidermal surfaces of GmSHN1-OE1 and GmSHN9-OE1 were covered with regions of high wax deposition.

**Figure 8:**
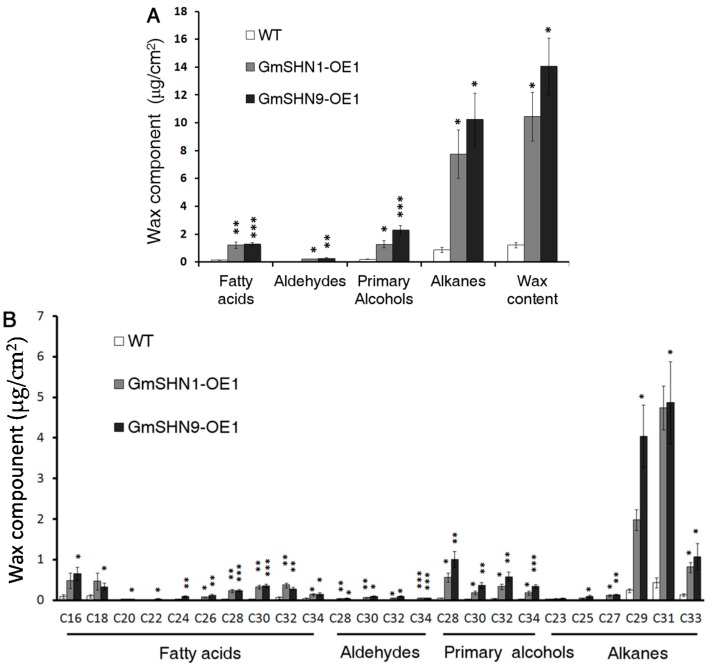
Analysis of leaf cuticular wax contents and components of GmSHN1-OE1, GmSHN9-OE1, and WT. (**A**) Quantitative analysis of wax contents and components in Arabidopsis leaves; (**B**) Quantitative analysis of constituents of fatty acids, aldehydes, primary alcohols, and alkanes in Arabidopsis leaves. The results are given as mean values with ±SD (*n* = 4). Significance levels between WT, GmSHN1-OE1, and GmSHN9-OE1 were assessed by Student’s *t* test (* *p* < 0.05, ** *p* < 0.01, *** *p* < 0.001).

Leaf epicuticular wax content and component were further quantitatively analyzed by using gas chromatography–mass spectrometry (GC-MS). Our results showed that total epicuticular wax contents were increased 7.8-fold in GmSHN1-OE1 and 9.9-fold in GmSHN9-OE1 when compared with WT (Figure 8A). Alkanes accounted for more than 70% of the total leaf wax content in the two transgenic plant leaves. Primary alcohols and fatty acids were also significantly increased in leaves of GmSHN1-OE1 and GmSHN9-OE1. Additional data are given in Table S1. Further detailed analysis showed that the most prominent components are hentriacontane (C31 alkane) and nonacosane (C29 alkane), which accounted for more than 60% of the total leaf wax content (Figure 8B). The content of nonacosane (C29 alkane) led to the main difference between total wax contents of GmSHN1-OE1 and GmSHN9-OE1. Also, there were increases in contents of primary alcohols, including 1-octacosanol (C28), 1-triacontanol (C30), and 1-dotriacontanol (C32). The increase of C28 primary alcohol was most notable. Furthermore, contents of octacosanoic (C28), triacontanoic (C30), and dotriacontanoic (C32) acids were notably increased. Additionally, long chain fatty acids, hexadecanoic (C16) and octadecanoic (C18) acids, were significantly increased in leaves of both transgenic plants.

### 2.6. Overxpression of GmSHN1 and GmSHN9 Affects Cuticle Property and Alters Cuticle Composition in Transgenic Arabidopsis Leaves

**Figure 9:**
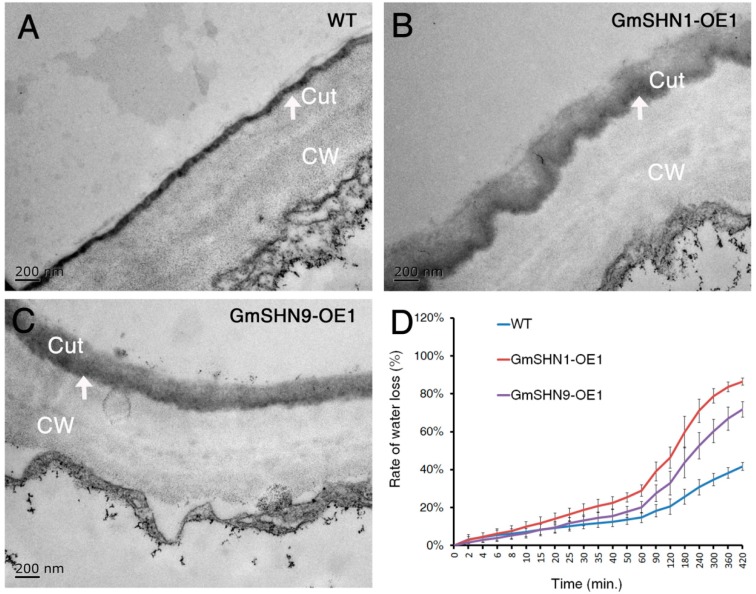
TEM ultrastructure of cuticle layer and rate of water loss. (**A**–**C**) TEM images of 4-week-old leaf epidermal cell sections of GmSHN1-OE1, GmSHN9-OE1, and WT. A thicker but loosely packed layer of electro dense material (the arrows) can be observed in GmSHN1-OE1 and GmSHN9-OE1 epidermal cells. Cuticle layers were shown by arrows. Cut, cuticle layer; CW, cell wall; Scale bars, 200 nm; (**D**) Rate of water loss of 4-week-old dark-adapted Arabidopsis rosette tissues of GmSHN1-OE1, GmSHN9-OE1, and WT. Four rosette tissues (root detached) were weighed at each time. The results are the mean values of three independent assays, and error bars indicate ±SD.

To Further investigate the effects of overexpression GmSHN1 and GmSHN9 on the cuticle of Arabidopsis, leaf sections of 4-week-old GmSHN1-OE1, GmSHN9-OE1, and WT plants were examined by transmission electron microscopy (TEM). Differences in cuticle deposition were obvious between transgenic and wild-type plants. The cuticle membrane of WT (Figure 9A) was compact and can be divided into two layers: an electron-translucent outer layer and an osmium-dense inner layer. GmSHN1-OE1 (Figure 9B) and GmSHN9-OE1 (Figure 9C) have a significantly thicker, but loosely packed and irregular layer of osmium-dense material on the outer cell wall of leaf epidermal cells than that of WT.

Cuticle has a major physiological function in the restriction of water loss [31]. We then compared rate of water loss using 4-week-old dark-adapted Arabidopsis rosette tissues. Our results showed that the water loss rates of the 4-week-old dark-adapted Arabidopsis rosette tissues were increased in GmSHN1-OE1 and GmSHN9-OE1 when compared with WT (Figure 9D). The experiments were repeated for three times on different days, and the results represent mean values ±SD from three independent experiments.

**Figure 10:**
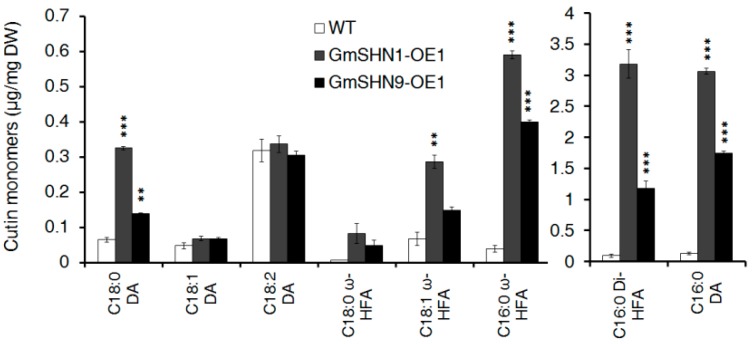
Leaf cutin monomer composition of the GmSHN1-OE1 and GmSHN9-OE1 compared with the WT. DA, dioic acid; HFA, hydroxyl fatty acid; Di-HFA, di-hydroxyl fatty acid. The results are given as mean values and error bars means SD (*n* = 3). Significance levels between GmSHN1-OE1, GmSHN9-OE1, and WT were assessed by Student’s *t* test (* *p* < 0.05, ** *p* < 0.01, *** *p* < 0.001).

To investigate whether the altered cuticle properties in GmSHN1-OE1 and GmSHN9-OE1 leaves are associated with the changes in cutin composition and content, we analyzed cutin components and amounts of 4-week-old Arabidopsis rosette leaves by GC-MS. Total leaf cutin contents were 7.92 μg·mg^−2^ (dry weight) in GmSHN1-OE1, 3.95 μg·mg^−2^ in GmSHN9-OE1, and 0.69 μg·mg^−2^ in WT. These results showed that total cutin contents in GmSHN1-OE1 and GmSHN9-OE1 increased 11.4-fold and 5.7-fold, respectively, when compared with that in WT (Figure 10). Overexpression of GmSHN1 and GmSHN9 greatly increased the accumulation of hexadecane-1, 16-dioic acid (16:0 DA), 9(10),16-di-hydroxy-hexadecanoic acid (16:0 di-HFA), and 16-hydroxy-hexadecanoic acid (16:0 ω-HFA) in Arabidopsis leaves. There was also an obvious increase in the amounts of octadecane-1,18-dioic acid (18:0 DA), 18-hydroxy-octadecanoic acid (18:0 HFA), and 18-hydroxy-octadecenoic acid (18:1 HFA) in leaves of GmSHN1-OE1 and GmSHN9-OE1 when compared with WT. By contrast, the level of octadecadiene-1,18-dioic acids (18:2 DA) was the most abundant in WT Arabidopsis leaves and took up more than 40% of total cutin content. The accumulation of octadecene-1,18- and octadecadiene-1,18-dioic acids (18:1 DA and 18:2 DA) were not significantly changed in GmSHN1-OE1 and GmSHN9-OE1 when compared with WT. The significantly increased cutin accumulation in leaves of GmSHN1-OE1 and GmSHN9-OE1 indicates that both GmSHN1 and GmSHN9 play important roles in regulating cutin biosynthesis.

### 2.7. Overxpression of GmSHN1 and GmSHN9 Induces Expression of Wax and Cutin Biosynthesis Related Genes in Transgenic Arabidopsis Plants

**Figure 11:**
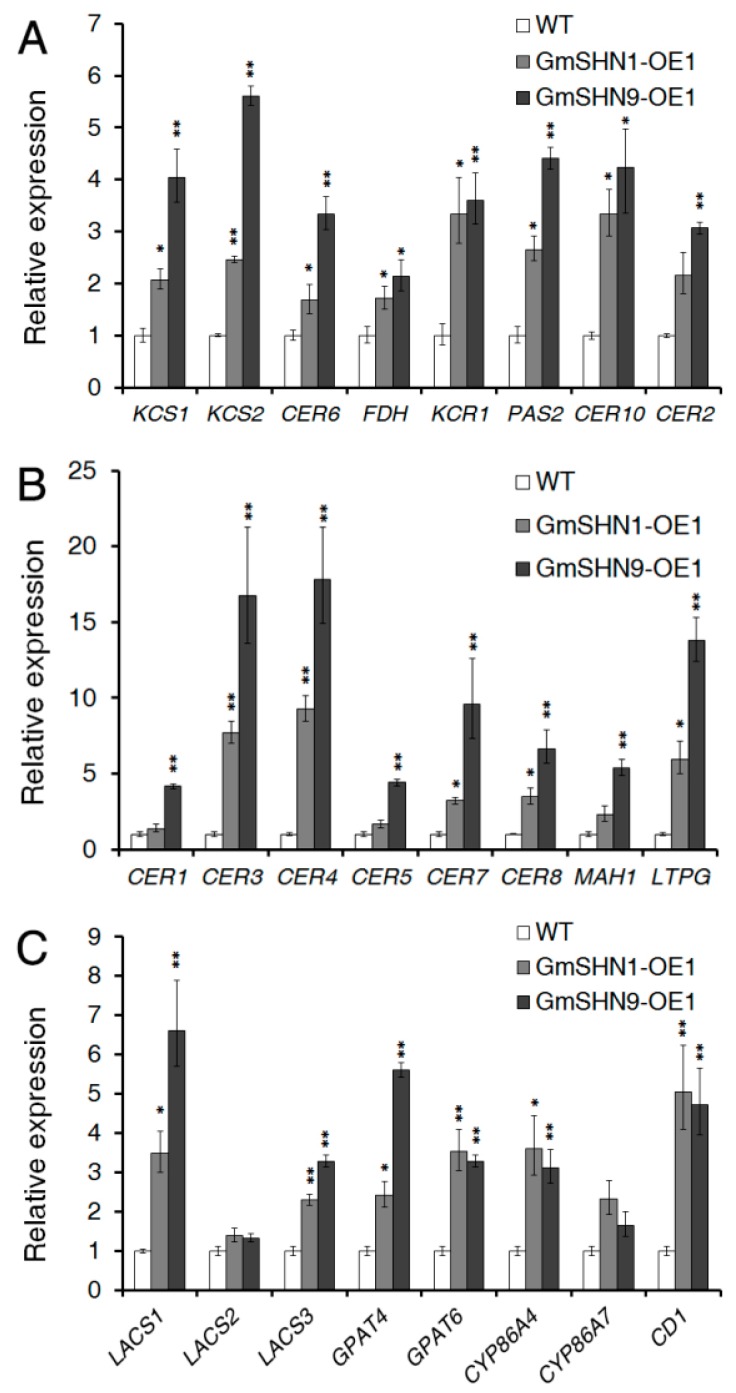
Expression of wax biosynthesis related genes in 6-week-old GmSHN1-OE1, GmSHN9-OE1, and WT Arabidopsis plants. (**A**) quantitative RT-PCR (qRT-PCR) analysis of the expression of genes involved in the fatty acid elongation process; (**B**) qRT-PCR analysis of the expression of genes involved in the biosynthesis of wax components; (**C**) qRT-PCR analysis of the expression of genes involved in the cutin biosynthesis pathway. Expression levels of each gene in WT were taken as 1. The Arabidopsis *ACT2* gene was used as the internal control for normalization of the template cDNAs. The results are given as mean values of three replicates and error bars represent ±SD (* *p* < 0.05, ** *p* < 0.01 *vs.* WT with two tailed Student’s *t* test).

To further investigate whether the increased wax production in transgenic plants is associated with the expression of genes involved in the wax biosynthesis pathway, the expression of 16 wax synthesis related genes was examined by quantitative RT-PCR (qRT-PCR). We found that eight genes which are involved in the fatty acid elongation process were up-regulated in GmSHN1-OE1 and GmSHN9-OE1 plants (Figure 11A), including *AtKCS1*, *AtKCS2*, *AtCER6*/*CUT1*, *AtKCS10*/*FDH* (encode ketoacyl-CoA synthetases), *AtKCR1* (encodes ketoacyl-CoA reductase), *AtPAS2* (encodes hydroxyacyl-CoA dehydratase), *AtCER10* (encodes enoyl-CoA reductase), and *AtCER2* (encodes CoA-dependent transferase). Among these genes, *AtKCS1*, *AtKCS2*, *AtKCR1*, *AtCER10*, *AtPAS2,* and *AtCER2* showed more than 2-fold up-regulation in both transgenic lines. *AtCER6*/*CUT1* showed a 3-fold up-regulation only in GmSHN9-OE1.

Eight genes involved in the biosynthesis and transportation of very-long-chain alkanes, alcohol, and aldehydes were differentially up-regulated, including *AtCER1*, *AtCER3*, *AtCER4*, *AtCER5*/*ABCG12* (encodes ABC half transporter), *AtCER7*(encodes exosomal exoribounclease), *AtCER8/AtLACS1* (encodes acyl-CoA synthetase), *AtMAH1*, and *AtLTPG* (Figure 11B). *AtCER3* and *AtCER4* showed more than 7-fold up-regulation in both transgenic plants. *AtCER1* and *AtCER5*/*ABCG12* were up-regulated only in the GmSHN9-OE1 plants.

Our data of GC-MS showed that the cutin contents and compositions were altered in the leaves of GmSHN1-OE1 and GmSHN9-OE1 plants. Therefore, we further investigated expression of genes involved in cutin biosynthesis and found that *AtLACS1*/*CER8*, *AtLACS3* (encodes acyl-CoA synthetases), *AtGPAT4*, *AtGPAT6* (encode glycerol 3-phosphate acyl transferases), *AtCYP86A4*, *AtCYP86A7* (encode fatty acid ω-hydroxylases), and *AtCD1* (encodes cutin synthase) were up-regulated in both GmSHN1-OE1 and GmSHN9-OE1 plants (Figure 11C). *AtLACS1*, which encodes an acyl-CoA synthetase that is involved in both long chain (C16) fatty acids for cutin synthesis and VLCFAs for wax biosynthesis, was greatly increased in both GmSHN1-OE1 and GmSHN9-OE1 (3.5-fold and 6.5-fold increases, respectively). *AtCD1*, which encodes a cutin synthase that is involved in the polymerization of cutin monomers, was significantly increased in GmSHN1-OE1 and GmSHN9-OE1 (5.1-fold and 4.8-fold increases, respectively). *AtLACS2*, which was reported to be directly regulated by *AtWIN1* [22], was only slightly increased GmSHN1-OE1 and GmSHN9-OE1.

## 3. Discussion

Previous studies have shown that various genes are associated with the complex regulatory network of wax biosynthesis [7]. Studies of some SHN AP2/ERF transcription factors have been reported to be involved in the wax biosynthesis process; however, the molecular mechanism of soybean SHN genes remains unclear. Our results showed that the homologs of AtSHNs in soybean may act as transcription factors since the sequence alignment analysis demonstrates that these GmSHNs have three conserved amino acid sequence domains in common with AP2/ERF transcription factors AtSHN1/SHN2/SHN3. Homologs of AtSHNs have also been found in rice and tomato [17,20].

### 3.1. GmSHN1 and GmSHN9 Differentially Regulate the Cuticular Wax Biosynthetic Pathway

GC-MS examination of total wax contents of GmSHN1-OE1 and GmSHN9-OE1 showed a 7.8-fold and 9.9-fold increase, respectively, when compared with that of WT, which were higher than the 6-fold enhancement in the Arabidopsis gain-of-function mutant [15] and 2-fold enhancement in the AtWIN1 overexpressors [16]. Among all the wax constituents, alkanes accounted for more than 70% of the total leaf wax content in transgenic plants. And C29 and C31 alkanes were the predominant constituents and took up more than 60% of the total wax contents in both transgenic lines. Additionally, content of C29 alkane in GmSHN9-OE1 was greatly higher than that in GmSHN1-OE1, which is the biggest difference between the two transgenic plants. Previous studies have indicated that odd numbered alkanes are the major wax component of mature leaves in species like Arabidopsis [9,31] and tomato [32]. VLC fatty acids and primary alcohols are the main constituents of cuticular wax in rice [17,33]. In addition, even homologous genes from different plants may lead to different wax compositions. For example, Arabidopsis SHN1/WIN1 increases wax accumulation mainly through elevating the constituent of C29 alkane [15], while OsWR1 up-regulates wax production mainly because of an enhancement in C30 fatty acid [17]. Distinct to the regulation of AtSHN1/WIN1 and OsWR1, GmSHN1 and GmSHN9 increase wax production mainly through changing the constituents of both C29 and C31 alkanes.

The Arabidopsis genes, such as *AtKCS1* [34], *AtKCS2* [35], *AtCER6/CUT1* [36], *AtFDH/KCS10* [37], *AtKCR1* [38], *AtPAS2* [39], *AtCER10* [40], and *AtCER2* [41], have been shown or proposed to encode enzymes or components of the VLC fatty acids elongation process. We found a 2-fold up-regulation of *AtKCS1*, *AtKCS2*, *AtKCR1*, *AtCER10*, *AtPAS2,* and *AtCER2* in both transgenic lines. Similarly, overexpression of AtSHN1/WIN1 in Arabidopsis stimulates expression of 3-ketoacyl-CoA synthase 1 [22]; overexpression of OsWR1 in rice elevates expression of six genes in the same pathway [17]; overexpression of OsWR2 in rice up-regulates 15 genes involved in wax biosynthesis pathway [18]. Thus both GmSHN1 and GmSHN9 may have a positive impact on the fatty acid elongation process which produces precursors for very-long-chain wax components. The increased total wax content of GmSHN1-OE1 (7.8-fold) and GmSHN9-OE1 (9.9-fold) further supported this supposition.

AtCER3 catalyzes the formation of alkane from VLCFA-CoA together with AtCER1 [42,43]. AtCER7 encodes an exoribonuclase involved in the degradation of a smRNA that negatively controls the transcription of AtCER3 [44]. We have examined a significantly up-regulated expression of AtCER3 and AtCER7 in both GmSHN1-OE1 and GmSHN9-OE1, which were consistent with the greatly increased accumulation of alkanes in both transgenic plants. Distinctive to the results that overexpression of AtCER1 dramatically increases the accumulation of alkanes [45], we did not observe an obvious change in AtCER1 expression. AtCER4 encodes an alcohol-forming fatty acyl-CoA reductase and its expression in yeast lead to the accumulation of C24 and C26 primary alcohols [46]. Our results showed that transcript levels of AtCER4 were at least 9-fold up-regulated in both transgenic plants, which was consistent with the increased production of primary alcohol. AtCER8/LACS1, which encodes an acyl-CoA synthetase that regulates both long chain (C16 and C18) fatty acids for cutin biosynthesis and VLCFAs for wax biosynthesis [18], were at least 4-fold up-regulated in both transgenic lines. In addition, Transcript levels of AtCER1, AtCER3, AtCER4, and AtCER7 were different between GmSHN9-OE1 and GmSHN1-OE1. These results indicate that GmSHN1 and GmSHN9 may differentially increase wax production through activating genes involved in decarbonylation and acyl reduction pathways.

### 3.2. GmSHN1 and GmSHN9 Are Involved in the Regulation of Cutin Biosynthesis, Especially Hydroxylation

Cutin is composed of interesterified (C16 and C18) dicarboxylic acids, hydroxyl fatty acids, and lesser amounts of glycerol. Previous studies show that mono- and poly- unsaturated dioic acids (18:1 DA and 18:2 DA) are the most abundant cutin constituents in Arabidopsis leaves [47]. Franke *et al.* reported that the octadecadien-1,18-dioic acid (C18:2 DA) was the most abundant cutin monomer and took up 20.97% of total cutin content [48]. In other reports, C18:2 DA content took up 40–60% of total cutin content [31,49,50,51]. Overexpression of *AtWIN1/SHN1* in Arabidopsis mainly altered the amounts of the dioic acids [22]. *OsWR1* (homolog of *WIN1/SHN1* in rice) overexpressors exhibited no significant alteration in cutin constituents at all [17]. Our results showed that 16:0 di-HFA, 16:0 DA, and 16:0 ω-HFA are greatly increased and became the main cutin constituents in transgenic plant leaves. Contents of 18:0 ω-HFA and 18:1 ω-HFA were also obviously increased in leaves of GmSHN1-OE1 and GmSHN9-OE1 than that of WT. The level of C18:2 DA was the most abundant in WT Arabidopsis leaves and took up more than 40% of total cutin content, while the accumulations of C18:2 DA were not significantly changed in GmSHN1-OE1 and GmSHN9-OE1 when compared with WT. CYP86A4 and CYP86A7, fatty acid ω-hydroxylases that use free long-chain (C16 and C18) fatty acids as substrates [52] and hydroxylate at the ω-position, were highly expressed in both GmSHN1-OE1 and GmSHN9-OE1. We further investigated the transcript levels of other cutin synthesis related genes and found that the overexpression of GmSHN1 and GmSHN9 in Arabidopsis up-regulated the expression of cutin related genes, and the transcript levels of *AtLACS1/CER8*, *AtLACS3*, *AtGPAT4*, *AtGPAT6*, and AtCD1 were induced to high levels [1,53,54,55]. Similarly, five genes known or likely involved in cutin biosynthesis were induced by AtWIN1/SHN1, including *AtLCAS2*, *AtCYP86A4*, *AtCYP86A7*, *AtGPAT4*, and *At1g12570* [22]. Taken together, it is clear that GmSHN1 and GmSHN9 both play important roles in the biosynthesis of cutin, especially in the hydration of C16 and C18 fatty acids, and that the regulatory functions of GmSHN1 and GmSHN9 in cutin biosynthesis pathways are somewhat different from that of AtWIN1/SHN1 and OsWR1.

Previous studies have shown that the biosynthetic and transport pathways of cutin and wax are coordinated [9]. *AtLACS1*, an acyl-CoA synthetase, is involved in both long-chain (C16) fatty acids for cutin synthesis and VLCFAs for wax biosynthesis [53]. In Arabidopsis, the ATP-binding cassette transporter gene *ABCG11* is required for both wax [56] and cutin export [57]. The biosynthetic and transport pathways of cutin and wax were differently regulated by GmSHN1 and GmSHN9. Overexpression of GmSHN9 leads to a higher wax accumulation (9.9-fold) in leaves of transgenic plants than overexpression of GmSHN1 (7.8-fold). Differing from the regulation of wax accumulation, the overexpression of GmSHN1 in Arabidopsis induces a higher increase in cutin content (11.4-fold) than induced content (5.7-fold) by overexpression of GmSHN9. Results of wax and cutin accumulation in GmSHN1-OE1 and GmSHN9-OE1 indicate that the regulatory emphasis of GmSHN1 and GmSHN9 on biosynthesis of wax and cutin are different.

### 3.3. GmSHN1 and GmSHN9 Are Involved in Regulation of Cuticle Property and Permeability

Cuticle is a hydrophobic layer that consists of a cutin polyester matrix filled with intracuticular waxes and covered with epicuticular waxes [5,6]. How the cutin monomers and wax affect cuticle property and permeability is still not very clear. Previous studies show that the amount of cuticular wax and cutin does not always directly associate with cuticle permeability [6,15,19]. The *wax2* and *wdl1* mutants had no obvious changes in leaf cutin content and composition, but displayed greatly altered cuticle ultrastructures [58,59]. *TaSHN1* (homolog of *WIN1/SHN1*) overexpressors increased wax accumulation and increased cuticle permeability [19]. Our results of TEM analysis showed that GmSHN1-OE1 and GmSHN9-OE1 leaves have a significantly thicker, but loosely packed cuticle layer when compared with WT. This observation is consistent with the increased total cutin contents and altered cutin composition.

Rates of water loss are increased in 4-week-old Arabidopsis rosette tissues of GmSHN1-OE1 and GmSHN9-OE1 compared to the wild-type, which can be interpreted as increased cuticle permeability of water. The increased cuticular permeability of the GmSHN1-OE1 and GmSHN9-OE1 transgenic plants is consistent with the loosely packed and irregular ultrastructure of the cuticle layer. Furthermore, the increased cuticle permeability is similar to that of *AtSHN1* [15] overexpressors and *TaSHN1* [19] overexpressors, but different from that of transgenic lines overexpressing *OsWR1* [17] and *SlSHN3* [10]. These results suggest that homologous genes of WIN1/SHN1 from different species may act differently on cuticular permeability. The mechanism of how wax and cutin composition affect ultrastructure of GmSHN1-OE1 and GmSHN9-OE1 leaf cuticle layers is an interesting subject for future research.

### 3.4. GmSHN1 and GmSHN9 Are Involved in Regulation of Leaf Development

Cell proliferation and cell expansion together control the morphology development of leaf blades. The overexpression of AtLNG1 and AtLNG2 was reported to regulate longitudinal cell elongation and cause narrow leaf blades with long petioles [28]. Our results showed that transcript levels of *AtLNG1* and *AtLNG2* were greatly up-regulated in GmSHN1-OE1 and GmSHN9-OE1. Leaf phenotype of GmSHN9-OE1 also exhibited long petioles with slender and curly leaves, which was not obvious in GmSHN1-OE1. Previous research shows that *an3* mutants exhibit narrow-leaf phenotypes and that the overexpression of *AtAN3* leads to larger leaves than normal [60]. We found that AtAN3 was down-regulated in GmSHN9-OE1, which is consistent with the results in *an3* mutants. The transcript level of *AtAN3* was increased in GmSHN1-OE1, but we did not observe significant changes in the leaf shape of GmSHN1-OE1. We also analyzed the expression of other genes involved in leaf development, including *AtROT3*, *AtROT4*, *AtAN*, *AtTCP4*, *AtSWP*, and *AtGRF1* (Figure S1). However, transcript levels of these genes did not show significant changes in GmSHN9-OE1 and GmSHN9-OE1. The alterations in leaf morphology of GmSHN9-OE1 and GmSHN9-OE1 may be coordinately controlled by multiple related genes. Further research focusing on leaf development may offer new insight into the functions of soybean SHN proteins.

## 4. Materials and Methods

### 4.1. Plant Materials and Growth Conditions

All Arabidopsis plants used in this study are of the Columbia ecotype (col-0). Seeds were sterilized in 70% (*v/v*) alcohol for 10 min and sown on 1% sucrose and 0.8% agar plates containing MS medium [61]. Antibiotic selection was used where appropriate (10 mg/L glufosinate-ammonium for plants containing pCAMBIA3300-based constructs). After incubating at 4 °C in darkness for three days, Arabidopsis seedlings were grown in a growth room at 20 °C, 70% relative humidity, a 16/8 h light/dark cycle at a fluorescent light intensity of 100 µmol·m^−2^·s^−1^ [62].

Soybean (*Glycine max* (L.) Merrill, cv. Heinong 44) seeds were surface-sterilized in a hermetically sealed desiccator with chlorine gas produced by mixing 100 mL bleach (5.25% sodium hypochlorite) and 4 mL 12 N HCl for 16 h [63]. Sterilized seeds were germinated on MS media (3% sucrose, 0.8% agar, and MS salts and vitamins, pH 5.8) at 26 ± 2 °C, with a 16 h photo period of 100 µmol·m^−2^·s^−1^.

### 4.2. Identification and Analysis of GmSHN Genes

To identify all homologs of Arabidopsis SHN genes in soybean, we used three Arabidopsis SHN protein sequences as a query to search against the soybean phytozome database with BLASTP program [64]. Multiple sequence alignments were carried out by ClustalX 1.8. The GENEDOC program was used to modify the sequence alignments. The phylogenetic tree was constructed by using MEGA 6.0 with the neighbor-joining method (10,000 bootstrap replicates, as recommended by the program). The gene structures of GmSHNs were analyzed by comparing the gene sequences with AtSHNs gene sequences, and gene structures were mapped using Photoshop CS6 software (Availeble at: http://www.photoshop.com/).

### 4.3. RNA Extraction and Semi-Quantitative RT-PCR

Total RNA of soybean was extracted with plants TransZolTM reagent (TransGen Biotech, Beijing, China) according to the manufacturer’s instructions, treated with RNase free DNase (TaKaRa, Dalian, China) to remove the genomic DNA contamination. The first-strand complementary DNA (cDNA) was synthesized from the total RNA (2 µg) in a 20 µL volume with M-MLV reverse transcriptase (Promega, Madison, WI, USA).

For organ-specific expression analysis, we collected roots, stem, leaves, flowers, and pods separately from 10-week-old adult soybean plants to measure transcript levels of GmSHNs in various organs. The RNA extraction method was described above. The soybean housekeeping gene ACTIN2 (GmACT2) was employed for normalization of samples, and primer sequences are as follows: Forward (5′–3′), AATTCACGAGACCACCTACAAC; Reverse (5′–3′), TGAGCCACCACTAAGAACAATG. The corresponding specific primers are listed in the Table S2. The profiles of reverse transcription-polymerase chain reaction (RT-PCR) were the following: 94 °C for 4 min, 33 and 28 cycles of 94 °C for 30 s, 55–60 °C for 30 s, 72 °C for 45 s for GmSHNs RT-PCR products and GmACT2 products, respectively, and ending with an elongation step of 5 min at 72 °C.

### 4.4. Plasmid Construction

To obtain the open reading frames of GmSHN1, GmSHN2, GmSHN3, GmSHN4, GmSHN5, GmSHN1, GmSHN7, GmSHN8, GmSHN9, and GmSHN10, PCR was performed using the primers with appropriate restriction enzyme sites (Table S2). The full cDNA sequences obtained from the soybean database were sequenced, and then directly cloned into a pCAMBIA3300 vector under the control of a 35S promoter from the cauliflower mosaic virus, resulting in pCAMBIA3300-SHN1/SHN2/SHN3/SHN4/SHN5/SHN6/SHN7/SHN8/SHN9/SHN10, respectively. All vectors were validated by digestion of restrictive enzymes and sequencing.

### 4.5. Plant Transformation and Selection

The vectors constructed above were separately transformed to Agrobacterium tumefaciens strain GV3101 via electroporation. Plant transformation was done in Arabidopsis using the floral dipping method as described [65]. The T1 transgenic plants were screened by spraying with 0.05% (*v*/*v*) phosphinothricin (ppt) solution and confirmed by PCR. The T2 seeds were grown on MS agar plates containing 10 mg/L ppt and the transgenic lines with a 3:1 (resistant: sensitive) segregation ratio were selected to produce T3 seeds. The T3 lines displaying 100% ppt resistance were considered homozygous and used for further experiments.

### 4.6. Physiological Measurements

To measure the chlorophyll contents (Total Chl, Chl a, and Chl b), 4-week-old leaves were excised from well-watered WT and transgenic Arabidopsis lines and then soaked in 80% (*v*/*v*) acetone in the dark for 48 h at room temperature. The extracts were measured spectrophotometrically with absorptions at 644 and 663 nm according to the methods reported previously [66].

To measure leaf fresh weight normalized area, leaves of well-watered 4-week-old transgenic lines and WT Arabidopsis were excised and weighted. ImageJ software 1.6 (Available at: http://rsb.info.nih.gov/ij/) was used to measure leaf areas through digital photos of leaves [45].

### 4.7. Quantitative Real-Time RT-PCR (qRT-PCR)

For gene expression analysis of transgenic Arabidopsis lines, total RNA was extracted from seedlings of 6-weeks-old WT and homozygous transgenic lines expressing GmSHN1 and GmSHN9. The Arabidopsis ACTIN1 (AtACT1) gene was used as an internal control for the normalization of the template cDNAs, and primer sequences are as follows: Forward (5′–3′), AGAGATTCAGATGCCCAGAAGTCTTGTT; Reverse (5′–3′), AACGATTCCTGGACCTGCCTCATCATAC. The cDNAs were amplified using TransStart Green qPCR SuperMix UDG (TransGen Biotech) in a 20 µL reaction. The thermal cycle of qRT-PCR was programmed as follows: 50 °C for 2 min; 95 °C for 10 min; 40 cycles of 95 °C for 5 s, 58 °C for 15 s, and 72 °C for 10 s [67,68]. The corresponding specific primers are listed in the Table S2.

### 4.8. Protein Localization Analysis

The coding regions of GmSHN1 and GmSHN9 were obtained by RT-PCR using primers without stop codons (Table S2) and then were put into a pUC18-35s-sGFP vector to form fusion proteins [69]. The genes were cloned into upstream of GFP and driven by the 35S promoter. The pUC18 plasmid containing the GFP driven by the CaMV 35S promoter was used as a negative control. The wild-type Arabidopsis mesophyll protoplasts were isolated as described [70]. The constructed vectors and the negative control vectors were introduced into the protoplasts prepared above, and the transient expression assay was performed by polyethylene glycol (PEG). Transfected protoplasts were incubated in the dark at 22 °C for 20–24 h, and then the cells were placed on ice for 2 h. Subcellular localizations were visualized by using an OLYMPUS FV1000 laser confocal microscope system with excitation at 488 nm and emission at 505–530 nm [18].

### 4.9. Environmental Scanning Electron Microscopy Analysis

To visualize the alteration in cuticular wax content, a Quanta 200 FEG (FEI) scanning electron microscope was used to analyze the images of Arabidopsis plant leaves without any form of preparation [71]. The adaxial side of 4-week-old rosette leaves of GmSHN1-OE1, GmSHN9-OE1, and WT plants were collected and directly placed onto the stub. The accelerating voltage of the ESEM was 15 kV.

### 4.10. Wax Extraction and Chemical Analysis

Cuticular waxes of leaves of 6-week-old plants were extracted by immersing them for 30 s in 20 mL of chloroform containing tetracosane at 60 °C as the internal standard [12]. Extracts were dried under nitrogen and derivated by heating at 70 °C for 1 h in 30 μL bis-*N*,*N*-(trimethylsilyl) trifluoroacetamide (Sigma-Aldrich, Santa Clara, CA, USA) and 30 μL pyridine (Fluka, Buchs, Switzerland). Residual derivatization reagents were evaporated by nitrogen before samples were dissolved in hexane. The quantitative composition was studied with a capillary gas chromatography (GC) (6890N; Agilent, Palo Alto, CA, USA) system coupled to a Pegasus IV time-of-flight mass spectrometer system (LECO). Helium was used as the carrier gas for GC at a flow rate of 1.0 mL·min^−1^ and the program was as follows: injection at 280 °C, held for 2 min at 50 °C, then was linearly ramped to 200 °C at a rate of 20 °C·min^−1^, held for 2 min at 200 °C, then was linearly increased to 320 °C at a rate of 2 °C·min^−1^, and held for 14 min at 320 °C. A DB-5MS capillary column (30 m × 0.25 mm × 0.25 μm film thickness (Agilent J&W ScientiWc, Boblingen, Germany) was used to separate the samples. Monomers of cuticular wax were identified from their electron ionization mass spectrometry spectra combining with related standards. Their quantities were expressed per unit of leaf surface area. Leaf areas were measured by ImageJ software 1.6 (Available at: http://rsb.info.nih.gov/ij/) using digital photos of leaves [45].

### 4.11. Water Loss Analysis

Four-week-old Arabidopsis plants (WT, GmSHN1-OE1, and GmSHN9-OE1) were dark acclimated for 3 h to close stomata. Rosettes leaves were detached from roots and inflorescence stems, and then placed in water in the dark for 1 h to soak enough water [72]. Excess water was removed by blotting and the rosette leaves were placed in open petri dishes in the dark at room temperature (22 °C), 50% relative humidity, and were weighed every 2 min in the first hour. Time intervals of measurement and observation were increased to 0.5 h for the next hour, and were then increased to 1 h for the 2–7 h. Finally, the rosette leaves were dried for two days at 60 °C to determine the final dry weight. Water loss rate was calculated as weight of lost water per unit fresh weight. Measurements were taken from four different Arabidopsis plants of WT, GmSHN1-OE1, and GmSHN9-OE1. The experiments were repeated for three times at different days.

### 4.12. Transmission Electron Microscope (TEM)

Three 4-week-old rosette leaves (cut into 1 mm^2^ segments) from different Arabidopsis plants of each genotype were used for transmission electron microscopy. Samples were fixed for 4 h with 2.5% glutaraldehyde in a 0.1 M sodium phosphate buffer (PBS, pH 7.2) at room temperature [18]. After fixation, the samples were rinsed three times with 0.1 M PBS with each time lasting for 30 min. Samples were then post-fixed with 1% osmium tetroxide (OsO_4_) at 4 °C overnight. After being rinsed in PBS, the samples were dehydrated through an ethanol series (30, 50, 70, 90 and 100), infiltrated with LR White Resin, and polymerized at 60 °C for 24 h [19]. TEM samples were cut into 70 nm thick using an LEICA ULTRACUT and mounted on 100-mesh copper grids. The samples were stained with 2% uranyl acetate and 0.5% lead citrate [16], and then examined under a JEM-1400 (JEOL, Tokyo, Japan) electron microscope at 80 kV.

### 4.13. Cutin Extraction and Chemical Analysis

For cutin analysis, rosette leaves of 4-week-old Arabidopsis plants were immersed into 85 °C isopropanol for 15 min. After cooling to room temperature, samples were subjected to the removal of all the soluble lipids with successive treatments of CHCl_3_:CH_3_OH (2:1, *v*/*v*), CHCl_3_:CH_3_OH (1:1, *v*/*v*), and CH_3_OH [73]. All the treatments were performed at room temperature and 100 rpm on an orbital shake for 24 h. Samples were dried under a fume hood overnight at room temperature, and then were further dried in a vacuum desiccator for 3–5 days. The dry residue of each sample was weighed and internal standards (25 μg of methyl heptadecanoate and 25 μg of pentadecalactone) were added to each tube. Samples were depolymerized for 2 h at 60 °C with 1.5 mL sodium methoxide, 0.9 mL methyl acetate, and 3.6 mL methanol. After cooling, the fatty acid methyl esters were extracted with 10 mL methylene dichloride and 1.5 mL glacial acetic acid, and then were washed twice with 0.5 M NaCl. Extracts were dried under nitrogen, and were dissolved with 100 μL of pyridine and 100 μL BSTFA. Heat extracts were dried at 100 °C for 2 h. After cooling, extracts were evaporated under nitrogen and were dissolved with 100 μL of 1:1 (*v*/*v*) heptane: toluene. GC-MS was programmed with helium carrier gas flow set at 0.7 mL/min under the same conditions described above for the wax analysis. The oven temperature was programmed with 15 °C/min from 80 to 200 °C and 2 °C/min from 200 to 300 °C. The quantification of cutin components were based on peak areas compared to areas of internal standards.

## 5. Conclusions

In the present study, we have identified 10 soybean SHN homologs and found that the GmSHN genes were differentially expressed in various soybean organs. The heterologous expression of each GmSHN gene caused different leaf phenotypes in Arabidopsis, and the overexpression of GmSHN1 or GmSHN9 greatly increased wax accumulation in Arabidopsis leaves mainly through up-regulating the contents of C29 and C31 alkanes. GmSHN1 or GmSHN9 regulate cutin biosynthesis mainly through hydroxylation. Overexpression of GmSHN1 or GmSHN9 also altered leaf cuticle untrastructure and permeability in transgenic Arabidopsis plants. Taken together, our results suggest that GmSHN1 and GmSHN9 may differentially regulate wax and cutin metabolic pathways, alter cuticle property, and affect the leaf development process.

## Supplementary Materials

Supplementary materials can be found at http://www.mdpi.com/1422-0067/17/4/587/s1.
