# Supplementary material for: Overexpression of the Transcription Factors GmSHN1 and GmSHN9 Differentially Regulates Wax and Cutin Biosynthesis, Alters Cuticle Properties, and Changes Leaf Phenotypes in Arabidopsis
Source: Int J Mol Sci. 2016 Apr 21;17(4):587. doi: 10.3390/ijms17040587 (PMC4849042; doi:10.3390/ijms17040587)
Supplement: Supplementary file 1 [file ijms-17-00587-s001.pdf]

Supplementary Material: Overexpression of the Transcription Factors GmSHN1 and GmSHN9 Differentially Regulates Wax and Cutin Biosynthesis, Alters Cuticle Properties, and Changes Leaf Phenotypes in Arabidopsis

Yangyang Xu, Hanying Wu, Mingming Zhao, Wang Wu, Yinong Xu and Dan Gu

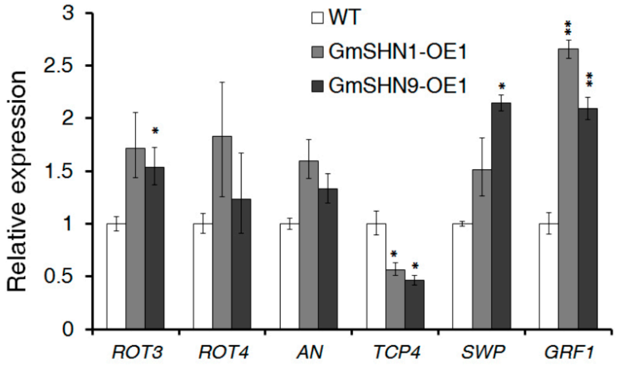

Figure S1. Expression of leaf development related genes in six-week-old transgenic and WT Arabidopsis. Expression levels of each gene in WT were taken as 1. The Arabidopsis *ACT2* gene was used as the internal control for normalization of the template cDNAs. The results are given as mean values of three replicates and error bars represent SD (n = 3). * $p < 0.05$, ** $p < 0.01$ *vs.* WT with two tailed Student's t test.

Table S1. Composition of cuticular wax on leaves of wild type, GmSHN1-OE1, and GmSHN9-OE1 transgenic plants.

Compound Class	Wild Type (μg/cm²)	GmSHN1-OE1 (μg/cm²)	GmSHN9-OE1 (μg/cm²)
Fatty acids	0.126 ± 0.014	1.213 ± 0.225	1.274 ± 0.124
Aldehydes	0.030 ± 0.006	0.212 ± 0.012	0.259 ± 0.059
Primary	0.182 ± 0.020	1.266 ± 0.268	2.278 ± 0.324
Alkanes	0.864 ± 0.198	7.74 ± 1.743	10.233 ± 1.892
Total	1.202 ± 0.201	10.436 ± 1.759	14.044 ± 2.020

The coverage of total wax and individual compound classes are given as mean values with ±SD (n = 4). Significance was assessed by Student's t test (* $p < 0.05$, ** $p < 0.01$, *** $p < 0.001$).

Table S2. Sequences of the primers used for transgenic Arabidopsis construction and identification of GmSHNs and cuticle related gene expression assays.

Gene Name	Sense Primer (5′–3′)	Sense Primer (5′–3′)
	Primers used for semi-quantitative RT-PCR amplifications	
GmSHN1	AGAGCAAGATAGTTCTGGCCA	AGCTCGGGATGATGGGGCTAATTC
GmSHN2	AGACAACATAGTTCTGACCAAC	CTCAATTCCTATTGAGAAGTTCC
GmSHN3	AGAACCCCAACAAACTCTTAAC	TCCATAACACTCTGTGGAAAAG
GmSHN4	AGACAAACTCTAACAACATCATGG	CTCGTGAATACCATAACGCTGTGT
GmSHN5	AGATAGTTACTGCTCTAACCAAC	CTCGTGAAAGCGAAATTGCGTTG
GmSHN6	GACCCCGAGAATTCCCACAT	AGAAGCTCCTGTCTCACCACTTG
GmSHN7	AGATCATGGTGCAAACAAGGAAG	CTCCTTCTTCAGGAAACATTTCC
GmSHN8	CATTTTGTAACAATAATACAATTAT	TACTAAACCGTTCAAAGACGTTTTC
GmSHN9	AGAAACACCCACCGCACAAAGCC	AGCTCAGTATTAGCCATGACACCAA
GmSHN10	AGATTTCTCACGCGCCATTAAC	CTCTGATGCTTAATTACCACCAA

Table S2. *Cont.*

Gene Name	Sense Primer (5′–3′)	Sense Primer (5′–3′)
Primers for construction and identification of transgenic Arabidopsis plants		
GmSHN1	TCATCTAGAGCAAGATAGTTCTGGCCA	GAGAGCTCGGGATGATGGGGCTAATTC
GmSHN3	TCATCTAGAACCCCAACAAACTCTTAAC	TCAGGATCCATAACACTCTGTGGAAAAG
GmSHN5	TCATCTAGATAGTTACTGCTCTAACCAAC	TCAGAGCTCGTGAAAGCGAAATTGCGTTG
GmSHN7	TCATCTAGATCATGGTGCAAACAAGGAAG	TCAGAGCTCCTTCTTCAGGAAACATTTCC
GmSHN8	TCACATTTTGTAACAATAATACAATTAT	CGTTACTAAACCGTTCAAAGACGTTTTC
GmSHN9	TCATCTAGAAACACCCACCGCACAAAGCC	CAGAGCTCAGTATTAGCCATGACACCAA
Primers for subcellular localizations		
GmSHN1	TGAGTCGACATGGTGCAATCAAGGAAA	GACCATGGAATTCCTATTCAGAAGTTC
GmSHN9	TGAGTCGACATGGTACAAGCAAAGAAG	GACCATGGATTAATTACTCGAACCACA
**Gene Name**	**Sense Primer (5′–3′)**	**Sense Primer (5′–3′)**
Primers used for quantitative RT-PCR amplifications		
AtCER1	GCTTAACAGGAACGGAGAGG	ATCACGACGCTTGTAGTTGC
AtCER2	AACGACAACGACACTTCAGC	GAGCCACTTCTCCACTGTGA
AtCER3	TTGGATGTTGCTTGTTTGGT	CCCATTTCTTGATGATGCAG
AtCER4	TTCCTCTGTGATCTTGATGCTGTT	TTGGTCCACGGTTTGATTGA
AtCER5	CGGTCTCGTCGCTTATGTAACA	TGCGGTCGCGTTAAGATCTC
AtCER6	AAATCTTCAACCCGAAATGG	GATCGCCTCTCCTCATTCTC
AtCER7	CAAAGGCGGTCCCTCAAAT	CCATCCTTGTGTTTTGTATCCTTCT
AtCER10	CTCATCTGGGTCTTCCTCGTTT	TCCAGGAGTCACGGGAAGAG
AtKCS1	GCGATTCTCCTCTCTAACCG	GGAAGAACCATCGGTCCTAA
AtKCS2	AACAGCTTCTTCAGGTTCAACCA	ACGGACGGTGTGGATGAGTT
AtFDH	TGTTCTGCCGTTATGCTCTC	GAGCTTCACCTCCAACTTCC
AtKCR1	ATCACCAGAGGGTTACGCAAA	CTGGAGGCATCTCTTGATGTTAAAT
AtPAS2	CCGATCTCTCCGCCATAAAA	CATCCAGCAAAGACGATCCA
AtMAH1	TGGCGATGCTAGGTTTTTACG	GAGGGATTTGGTGGAGCATTC
AtLTPG	TGGCACCAGCAGGAAAGTC	AGGAGACGGCCATCAAAGC
AtLACS1	AAAGGTTTTCCTCCGATTGATTC	CCACTCGACTCCCAGGTTCA
AtLACS2	CACGAGCGATGTGTTCTTCT	GTTTCTTGCGTATCAAGCCA
AtLACS3	AGAAGCGATGTTTGCACCATAA	TCCTCAATCACACGATCGAAGA
AtGPAT4	AACGGGACGACAGTTAGAGG	AAACTCCACCGATCACCTTC
AtGPAT6	CCTTCTCCGGTTTAGTGCTC	TTGCCTCCTTTACACGTCAG
AtCYP86A4	GCTTGGGCTCGGTTTAGAAGT	CATGAAACGCGAGAGGAGATC
AtCYP86A7	CGTCTCGTGCCGATTCTTG	GCGAATCCGTTCTCAGGAAA
AtCS1	TCGAAGGTGCATGGACATCA	GGGTCGTCCAATAGTGCTTTG
AtLNG1	CTCGTGGCCTACAGCAATCTG	CAGCGACCTCATTCCCAAGT
AtLNG2	GAACCCGCAACAATGACTCA	AAGCAGGTATCCTCTTGATCGAA
AtROT3	CGGCCTGTTACCTTCATGGA	ATCTGAGGAACGCGCCAAT
AtROT4	CTCTTCCATTTCTTGCACTTTTTTA	TTTGCGGTCGTGGTTCTG
AtAN	CGGTGCTGAAGGTCCACAAT	CCTGTTGCCTACTGGTGGATTC
AtAN3	GGAGGAGAGAAAGCGAGATAACAC	GACAACCCCCACCACAAAAC
AtTCP4	GCACGACGGTCTCACTCACAA	GATTCAGGCCATTGACTACACAAA
AtSWP	AGCGCCTGCCAAAGTGTCTA	TGGATAGGTGCCCTCGGTAA
